# Sepsis-induced selective loss of NMDA receptors modulates hippocampal neuropathology in surviving septic mice

**DOI:** 10.1371/journal.pone.0188273

**Published:** 2017-11-27

**Authors:** Shuibing Zhang, Xueqin Wang, Sha Ai, Wen Ouyang, Yuan Le, Jianbin Tong

**Affiliations:** 1 Department of Anesthesiology, the Third Xiangya Hospital, Central South University, Changsha, Hunan, P.R. China; 2 Center for Experimental Medicine, the Third Xiangya Hospital, Central South University, Changsha, Hunan, P.R. China; Universidade do Extremo Sul Catarinense, BRAZIL

## Abstract

**Background:**

Sepsis-induced neuroinflammation plays an important role in sepsis-related brain dysfunction. However, the molecules that are targeted during neuroinflammation resulting from sepsis-induced brain dysfunction remain unclear. Herein, we tried to investigate the expression and roles of NMDA receptor subunits during sepsis-related brain dysfunction.

**Methods:**

Sepsis was induced by cecal ligation and perforation (CLP) or by a single intraperitoneal injection of lipopolysaccharide (LPS, 8 mg/kg) in C57BL/6J mice. The NMDA receptor co-agonist D-serine was injected intraperitoneally for 3 days (500 mg/kg/day) to compensate for the loss of NMDA receptors. The behaviors of mice were tested in the Barnes maze and in the open field test. The mice were euthanized at the indicated time points. The brains were collected to detect the following: the levels of synaptophysin and NMDA receptor subunits GluN2A, GluN2B and GluN1 (by Western blot and RT-PCR); the number of CA1 neurons (by Nissl staining); neuronal activity (by p-CREB staining); neuroinflammation (by staining of Iba-1 and inflammatory factors IL-1β, TNF-α, NLRP3); and the levels of oxidative stress [by dihydroethidium (DHE)].

**Results:**

Sepsis selectively decreased the protein and mRNA levels of GluN2A, GluN2B and GluN1 but not the levels of synaptophysin or the neuronal number in the hippocampus of mice in either of the classic CLP-induced or LPS-induced sepsis models during the first 7 days after sepsis. Intraperitoneal injection of D-serine obviously limited the lipopolysaccharide-induced changes, including the impairment of learning and memory, the loss of NMDA receptor subunits, robust neuroinflammation, the levels of ROS stress and the decrease of p-CREB in the hippocampus of mice.

**Conclusion:**

These data suggest that the sepsis-induced selective loss of NMDA receptors modulates hippocampal neuropathology in the mice that survived sepsis, and the data show that NMDA receptors are potential targets for the improvement of brain dysfunction in sepsis survivors.

## Introduction

Sepsis is a life-threatening organ dysfunction due to a dysregulated systemic inflammatory response to infection with high incidence, high mortality, high cost and a low quality of life for survivors[1]. Sepsis occurs in up to 35.1 million people per year[2] and accounts for 10–50% of deaths among septic patients in Intensive Care Units. Therefore, sepsis is a major health concern worldwide. Previously, much attention was paid to how to help sepsis patients survive during the early stage of sepsis. With the recent progress in anti-infection treatments and goal-directed supportive treatments, early sepsis mortality has decreased[1]. However, new problems have appeared. The survivors of sepsis usually suffer physical, cognitive, and mental impairments[3,4,5]. Survivors especially suffer from sepsis-related brain dysfunction, which occurs in 8–70% of septic patients and is a common feature of sepsis[6,7,8,9]. The possible mechanisms of sepsis-related brain dysfunction include neuroinflammation, impaired cerebral microcirculation, oxidative stress, excitotoxicity, and blood-brain-barrier dysfunction[6,10,11,12,13]. However, the molecular mechanisms of sepsis-related brain dysfunction remain unclear.

NMDA receptors are ionotropic glutamate receptors, which are closely involved in synaptic plasticity, learning and memory[14]. GluN1 andGluN2A-D are the main NMDA receptor subunits[14]. Imamura et al. reported that LPS increased the immune-reactivity of GluN2B and interleukin-1 receptor and the activation of microglia in mice brains at 20 h after LPS treatment[15]. Savignac et al. found that a single injection of 0.75 mg/kg LPS decreased GluN2B levels in the frontal cortex at 28 h after LPS, but the treatment had no obvious effect on the levels of GluN1 or GluN2A[16].These data suggest that NMDA receptors could be the molecular targets of sepsis damage and that they may be involved in sepsis-related brain dysfunction in septic survivors. However, these studies detected only the changes in NMDA receptor levels in the early stage of sepsis. Both the changes in NMDA receptors in the late stage of sepsis and the roles of NMDA receptors in sepsis-related brain dysfunction remain unclear. In this study, we found that sepsis decreased the levels of GluN2A, GluN2B andGluN1 but not synaptophysin in the hippocampus of mice during the late stage of sepsis. The co-agonist of synaptic NMDA receptors D-serine[17] significantly improved the brain dysfunction and the neuropathology of the hippocampus in septic survivors. These data suggest that NMDA receptors are closely involved in the brain dysfunction of septic survivors, and they could be a potential target for improvement of sepsis-related brain dysfunction.

## Materials and methods

### Ethical statement

Experiments were performed in accordance with the guidelines for experimental animal use of Central South University. The protocol [LLSC (LA).2015–018] was approved by the ethics committee of the 3rd Xiangya Hospital of Central South University.

### Animal experiments

C57BL/6J male mice (2 months, 20–25 g) were purchased from Central South University (Hunan, China) and were housed with a 12-hour day/night cycle with free access to food and water in a temperature-controlled room at 24±1°C. All mice were acclimated to the environment for seven days before the experiments.

#### Experiment 1

To detect the effects of sepsis on the expression of NMDA receptors and the synaptic protein synaptophysin, fifty-six mice were randomly divided into two groups. Thirty-one mice were used for the cecal ligation and perforation (CLP) model, the others were used for the LPS model. Mice for the CLP model were further divided into sham and CLP groups. Mice in the sham group received a surgery of the abdominal wall and were sacrificed at 7d after surgery under sevoflurane anesthesia (n = 5). Mice in the CLP group received a CLP surgery and were sacrificed at 6 h, 1d, 3d or 7d after surgery under sevoflurane anesthesia (n = 5 per time point). Six mice died during the first 3d after CLP surgery.

Mice for the LPS model were further divided into control and LPS groups. Mice in the control group received a single intraperitoneal injection of saline (n = 5). Mice in the LPS group received a single intraperitoneal injection of 8 mg/kg LPS[18], and no animals died in this model. In addition, the mice in the LPS group were sacrificed at 6 h, 1d, 3d or 7d after LPS injection (n = 5 per time point).

#### Experiment 2

To detect the effects of NMDA receptor changes on sepsis-related brain dysfunction and neuropathology, one hundred seventy mice (2 months old) were randomly divided into 3 groups: (1) control: mice that received normal saline intraperitoneal injections for 3 days (n = 12 for behavior, n = 10 for the mechanism study); (2) LPS+NS: mice that received one intraperitoneal injection of 8 mg/kg LPS[18] and saline injections for 3 days (n = 12 for behavior, n = 62 for the mechanism study); (3) LPS+D-serine: mice that received one intraperitoneal injection of 8 mg/kg LPS and injections of D-serine for3 days (500 mg/kg/day) [19](n = 12 for behavior, n = 62 for the mechanism study).

### Cecal ligation and perforation (CLP) surgery

The classical murine model of CLP was generated according to previously reported procedures[20]. Briefly, the abdomen of mice was shaved and thoroughly cleaned with complex iodine under inhaled sevoflurane anesthesia, and surgeries were conducted on an electric blanket. An incision of 1.5 cm was made at the midline of the abdomen. The cecum was exposed, ligated below the ileo-cecal valve, and then punctured once with a sterile 20-gauge needle. The cecum was then squeezed to expel a small amount of fecal material and returned to peritoneal cavity. Subsequently, the abdominal cavity was closed using aseptic 5–0 surgical sutures. The body weight, rectal temperature and survival of mice were carefully monitored every 8 hours before the endpoints. After the surgery, all mice were immediately placed in a warm environment and were administered a subcutaneous injection of pre-warmed saline (1mL) every 6 hours for fluid resuscitation[21]. Softened food was placed onto the bedding for mice to easily access. Buprenorphine (0.1 mg/kg) and bupivacaine (3 mg/kg) were injected subcutaneously for postoperative pain control[22]. All measures were taken to relieve the suffering of the animals. The criteria used for humane endpoints were as follows: hunched posture, decreased activity, labored breathing, loss of appetite and piloerection[23]. Experiments were stopped if mice met the above criteria. Mice in the sham surgery group were handled in similar manner, but the cecum was neither ligated nor punctured.

### Drug administration

D-serine is a co-agonist of synaptic NMDA receptors and competitively inhibits the phosphorylation of AMPK by reducing the binding of ATP to the α subunit of AMPK[17]. According to the reported dose and method, a small dose of D-serine was used. Briefly, D-serine (Sigma-Aldrich, America, S4250) was prepared with normal saline and was intraperitoneally injected for three days (500 mg/kg)[19,24]. LPS (Sigma-Aldrich, America, L2880) was diluted with normal saline and was intraperitoneally injected (8 mg/kg, once per mouse)[18]. The body weight, rectal temperature and survival of mice were carefully monitored before the endpoints or behavioral tests. During the first 72 hours, mice were monitored every 8 hours and then once daily until the behavioral tests or endpoints. After the injections, all mice were immediately placed in a warm environment and were administered a subcutaneous injection of pre-warmed saline (1 mL) every 8 hours for fluid resuscitation during the first three days[21]. Softened food was placed onto the bedding for mice to easily access. All measures were taken to relieve the suffering of the animals. The criteria used for humane endpoints were as follows: hunched posture, decreased activity, labored breathing, loss of appetite and piloerection[21]. Experiments were stopped if mice met the above criteria.

### Barnes maze test

Mice were tested as previously described[25]. Briefly, mice were trained to locate the escape hole on a Barnes maze four times per day after LPS injection on days 8 to 11 (3 min per trial and 15 min between each trial). The number of incorrect holes investigated (termed errors) and the latency to the target hole during each trial were recorded. The platform surface was cleaned with 75% ethanol before each trial to remove the odor cues. The test was performed by a technician who was blinded to experimental design.

### Open field test

The open field test was used to evaluate anxiety-like behavior and locomotor activity. The arena was made of five light gray plastic panels (50 cm × 50 cm × 50 cm) and it was divided into two areas: the central area including nine 10 cm × 10 cm grids and the peripheral area including sixteen 10 cm × 10 cm grids approaching the walls. The behavioral test began when a mouse was gently placed in the center arena facing the same direction in each test and was continued for 5 minutes. A video tracking system (Logitech, Suzhou, China) tracked the performance of each mouse. The arena was thoroughly cleaned with 75% ethanol between each session and was not used until the odor volatilized. The following variables were calculated by Smart Junior software[26] during each 5 minutes: total locomotor distances (including the central and the peripheral areas), and the ratio of locomotion time in the central area to the total locomotion time. Data were analyzed by a trained student who was blinded to the experimental conditions.

### Immunostaining

Mice were anesthetized with inhaled sevoflurane anesthesia and perfused transcardially with 0.01 M phosphate-buffered saline (PBS). The brains were removed. One hemisphere of each brain was used for immunostaining, and the other was used for Western blotting. The hemisphere used for immunostaining was fixed in 4% paraformaldehyde overnight at 4°C. After dehydrating with sucrose, the brains were embedded in OCT, and transverse sections of the brain (20 μm) were serially cut using a cryostat. Five sections of hippocampus (at levels of -1.22, -1.42, -1.62, -1.82, and -2.02 mm relative to the bregma)[27] were chosen for immunostaining of Iba-1 and p-CREB. After three washes in 0.01 M PBS, these sections were blocked with 5% BSA in 0.01 M PBS plus 0.3% Triton X-100 for one hour at room temperature and then incubated in primary antibodies (rabbit polyclonal antibody to Iba1: 1:1000, Wako, Japan, catalog number 019–19741; rabbit polyclonal antibody to p-CREB:1:500, Cell Signaling, USA, catalog number 9198) at 4°C overnight. After washing three times in 0.01 M PBS, the sections were first incubated in secondary antibodies (1:200) for two hours at room temperature and then washed three times. Finally, the sections were incubated in ABC (1:200) for one hour at room temperature, washed three times, and visualized using a DAB kit. Images in regions of the CA1 and the dentate genus (DG) in all sections were acquired under the same magnification (40x objective lens) and same light intensity by an author who was blinded to treatments. Based on the Iba1 staining, the percent of activated microglia in the CA1 and DG were also determined using the method reported by Cerbai F et al[28]. According to the report, a resting microglia was defined as when the cell body was small and round and the branches were thin, highly ramified, and equally distributed around the cell body. In contrast, an activated microglia was defined as when the cell body was bigger, pleomorphic bi- or tri-polar, or spindle/rod-shaped, and the branches were shortened, twisted or displayed no ramification[28]. Based on the p-CREB staining, the relative mean optical density was calculated using ImageJ. Data were analyzed by a trained technician who was blinded to experimental conditions.

### Western blotting

Western blotting was used to detect the expression of GluN1, GluN2A, GluN2B, synaptophysin and GAPDH in the hippocampus. Briefly, frozen hippocampus tissue was homogenized in a lysis buffer containing a protease inhibitor cocktail (Roche, Germany, catalog number: P8340) and phenylmethanesulfonyl fluoride (PMSF, Sigma, USA, catalog number: p7626). The quantity of protein in the samples was determined using a BCA protein assay kit (CWbio, China) according to the manufacturer’s instructions. Equal amounts of protein samples (X μg/lane) were separated by sodium dodecyl sulfate polyacrylamide gel electrophoresis (SDS-PAGE) and transferred to polyvinylidene fluoride membranes. After washing, the membranes were blocked with 10% skim milk in TBST buffer for 1 h and then incubated with primary antibodies (rabbit monoclonal antibody to GluN1: 1:1000, Cell Signaling Technology, USA, catalog number 5704; rabbit polyclonal antibody to GluN2A: 1:1000, Abcam, USA, catalog number ab169873; rabbit polyclonal antibody to GluN2B: 1:2000, Proteintech, USA, catalog number 21920-1-Ap; rabbit polyclonal antibody to synaptophysin:1:2000, Proteintech, USA, catalog number 17785-1-AP; rabbit polyclonal antibody to GAPDH: 1:2000, Proteintech, USA, catalog number 10494-1-AP) overnight at 4°C. After three washes, the membranes were incubated with a secondary antibody (1:2000) at room temperature for 2 h. Finally, the proteins were visualized using an enhanced chemiluminescence detection kit (CWbio, China), and the intensity of each band was quantified by densitometry. Relative expression levels of protein were normalized and are presented as the ratio of the target protein (GluN1, GluN2A, GluN2B, synaptophysin) to GAPDH.

### Real-time PCR examination

Total RNA was isolated using the Trizol extraction method according to the manufacturer's instructions (Invitrogen, USA, catalog number: CA02008). The quality of RNA was evaluated by comparing the optical densities at 260 nm and 280 nm. The extracted RNA was reverse-transcribed into cDNA using a Prime Script reverse transcription-PCR kit (GeneCopoeia, China, catalog number: QP001) for quantitative PCR. The primers (Table 1) for all assayed genes were determined using reported sequences and are listed in Table 1. The mouse GAPDH gene was used as an internal control. Data were analyzed and quantified using the 2^− ΔΔ^Ct method.

**Table 1:** Primers used for quantitative real-time PCR.

Target gene	Primer	Sequence (5′–3′)
NLRP3	Forward	ATTACCCGCCCGAGAAAGG
	Reverse	TCGCAGCAAAGATCCACACAG
IL-1β	Forward	GCCCATCCTCTGTGACTCAT
	Reverse	AGGCCACAGGTATTTTGTCG
TNF-α	Forward	ATGCACCACCATCAAGGACTCAA
	Reverse	ACCACTCTCCCTTTGCAGAACTC
GluN2A	Forward	TGCTCATCACCTCATTCTTCTC
	Reverse	GATTGACCTCGCTCTGCTC
GluN2B	Forward	CACAAACATCATCACCCACAC
	Reverse	TTGACTTCTCTGTGCCCTTC
GluN1	Forward	GCTGTACCTGCTGGACCGCT
	Reverse	GCAGTGTAGGAAGCCACTATGATC
SYN	Forward	CTGCGTTAAAGGGGGCACTA
	Reverse	ACAGCCACGGTGACAAAGAA
GAPDH	Forward	GGTGAAGGTCGGTGTGAACG
	Reverse	CTCGCTCCTGGAAGATGGTG

### Reactive oxygen species (ros) detection

The level of reactive oxygen species in brains was detected using a previously reported method[29]. Briefly, the oxidation-sensitive fluorescent marker dihydroethidium (DHE, Beyotime Biotechnology, China) was solubilized in 50% DMSO in 10 mmol/L phosphate buffer (5 mg/mL). Normal and sepsis-surviving mice were injected with DHE (9 mg/kg) intraperitoneally 24 hours and 21.5 hours before killing. The next day, mice were anesthetized and transcardially perfused with 4% paraformaldehyde. Brains were removed, post-fixed with 4% paraformaldehyde for 24 hours, dehydrated with sucrose, and cut into 25-μm sections. Sections (at levels of -1.22, -1.42, -1.62, -1.82, and -2.02 mm relative to the bregma)[27] were rinsed with PBS for 5 minutes. DHE produces the fluorescent compounds ethidium(Et) and 2-hydroxyethidium when oxidized by inner ROS. The Et-positive fluorescence images were captured, and the relative mean fluorescence was calculated using ImageJ. Data were analyzed by a trained student who was blinded to experimental conditions.

### Statistical analysis

The Barnes maze data are presented as the mean ± standard error (mean ± SEM) and were analyzed using repeated measures ANOVA with the treatment as the between-subjects factor and the measure of time as the within-subjects factor, followed by the Bonferroni test. Biochemical data are presented as the mean ± standard deviation (mean ± SD) and were analyzed using two-way ANOVA followed by the Bonferroni test. Data analyses were performed using SPSS 18.0 for Windows, and *P*<0.05 was considered statistically significant.

## Results

### Sepsis-induced selective loss of NMDA receptors in the hippocampus

**Fig 1:**
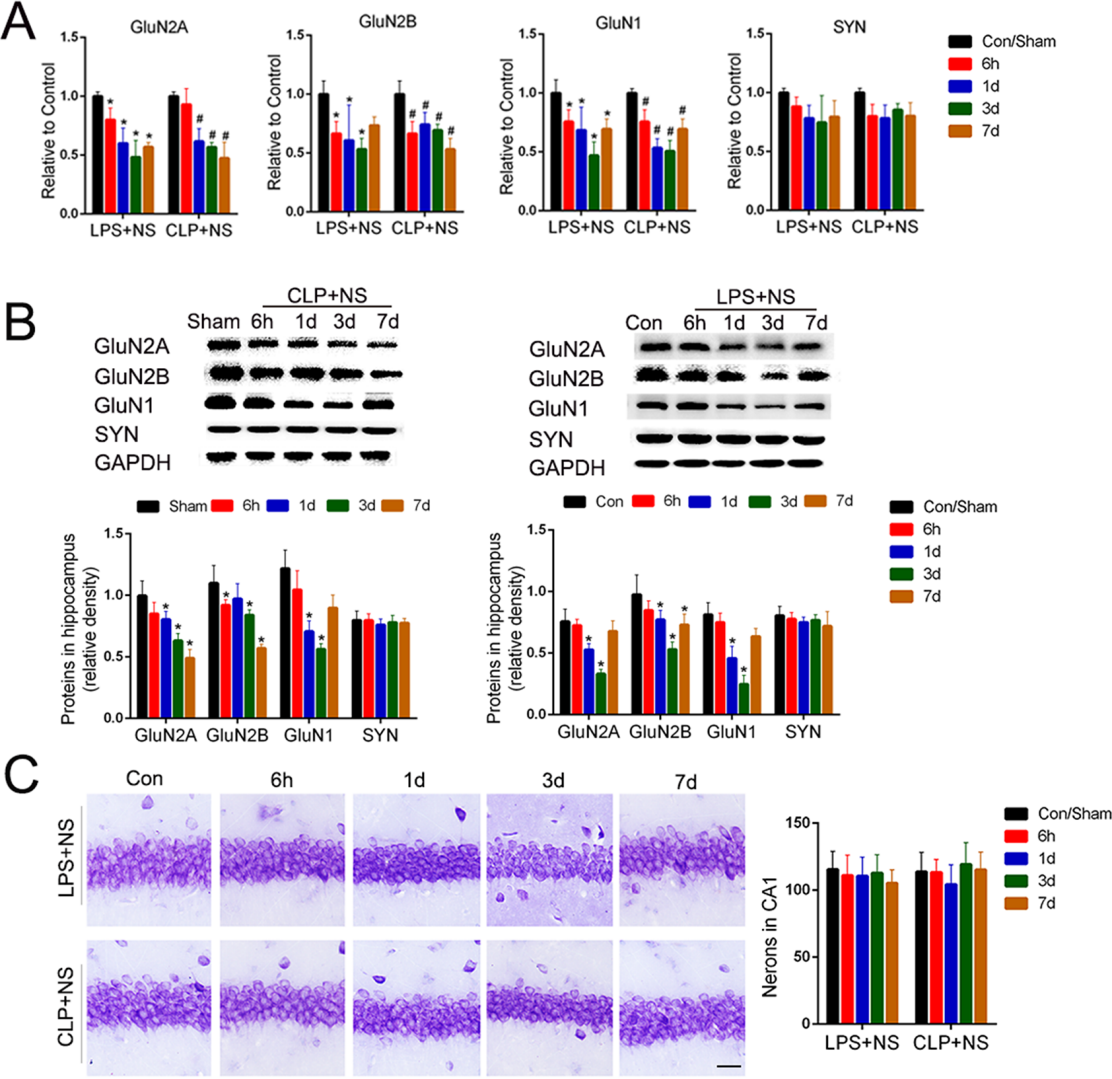
Sepsis-induced selective loss of NMDA receptors in the hippocampus. (A) Quantitative RT-PCR analysis of mRNA levels of NMDA receptor subunits (GluN2A, GluN2B, and GluN1) and synaptophysin in the hippocampus of sepsis model mice induced by cecal ligation and puncture (CLP) or by intraperitoneal injection of lipopolysaccharide (LPS, 8 mg/kg). Data are expressed as the mean ± S.D. * or ^#^*P*<0.05 vs. control or sham group, n = 5 per time point per group. (B) Representative Western blot analysis of NMDA receptors subunits (GluN2A, GluN2B, and GluN1) and synaptophysin in the hippocampus of CLP and LPS models. Data are expressed as the mean ± S.D. *P<0.05 vs. control or sham group, n = 5 per time point per group. (C) Representative Nissl staining analysis of neurons in the hippocampal CA1 region of CLP and LPS mice, Bar = 100μm. Data are expressed as the mean ±S.D. There were no significant differences among these groups (*P*>0.05).

To detect the effects of sepsis on the expression of NMDA receptors during the late stage of sepsis, we detected the levels of NMDA receptor mRNA and protein in two types of classic sepsis models induced by CLP or by intraperitoneal injection of LPS. Compared to the normal control group, the mRNA levels of GluN2A, GluN2B, and GluN1in the hippocampus were all decreased to an extent during the first 7 days in the CLP and LPS models (*P*<0.05; Fig 1A). Consistent with the mRNA change, the protein levels of GluN2A, GluN2B, and GluN1 in the hippocampus in the CLP and LPS models were less than those of the normal control animals during the first 3 days (*P*<0.05; Fig 1B), but the protein levels were partly restored on the 7^th^day in the LPS model (*P*>0.05; Fig 1B). In contrast, the expression levels of the synaptic vesicle protein synaptophysin in the CLP and LPS models were not obviously changed relative to the normal controls (*P*>0.05; Fig 1A and 1B). The number of neurons in the CA1 was not obviously decreased in the CLP and LPS models during the first 7 days (*P*>0.05; Fig 1C). These data suggest a sepsis-induced selective loss of NMDA receptors in the hippocampus.

### Activation of NMDA receptors with D-serine noticeably improved the brain dysfunction in LPS-treated surviving mice

**Fig 2:**
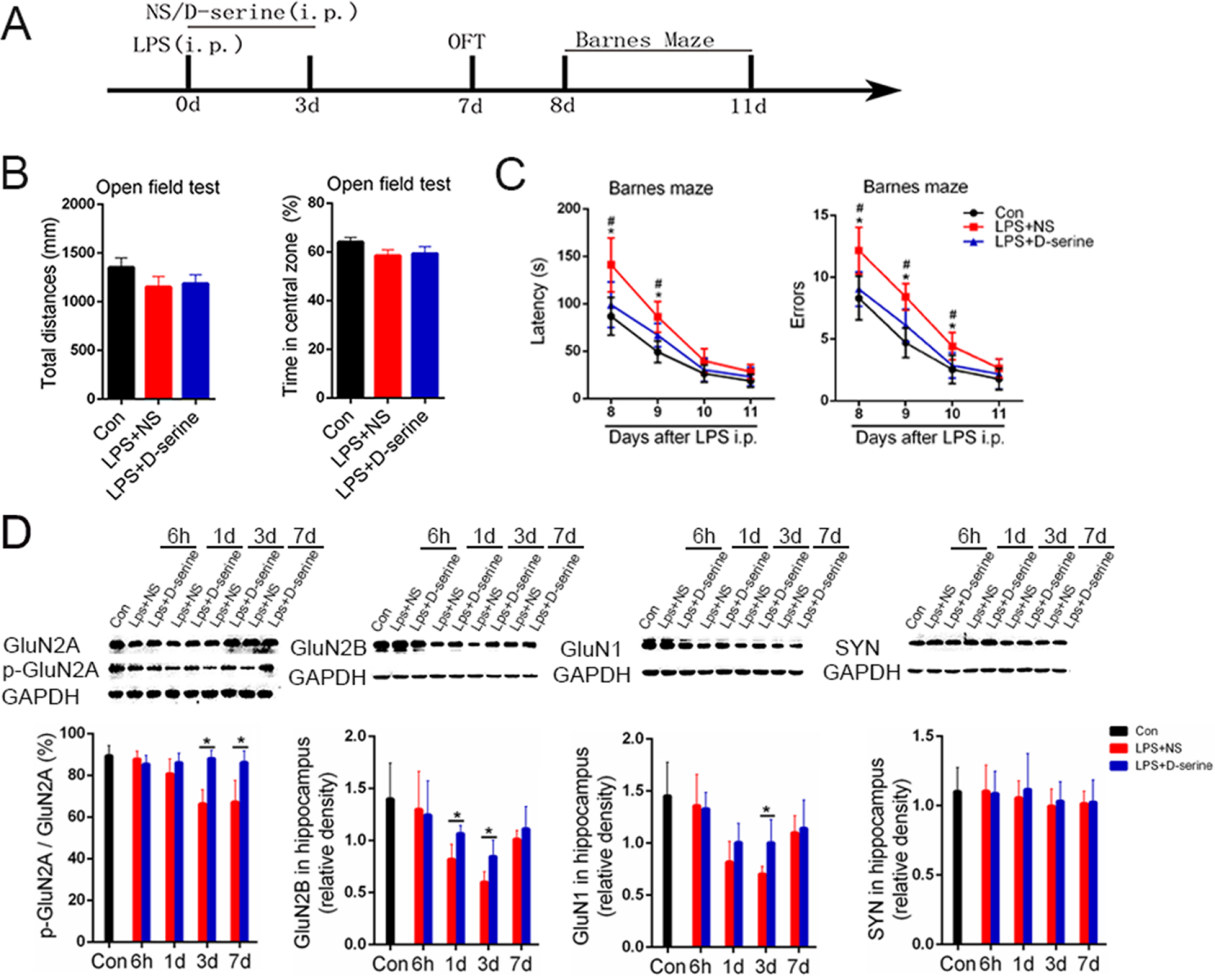
Activation of NMDA receptors with D-serine obviously improved the brain dysfunction and limited NMDA receptor loss in the surviving LPS-treated mice. (A) The schedule for drug administration and different behavioral tests. (B) The open field test results of the surviving sepsis mice. There was no significance between the time spent in exploring the central area and the total locomotor distances among the three groups (n = 12/group). (C) The Barnes Maze test results of the surviving sepsis mice. Data are expressed as the mean ± S.E.M.*P<0.05 LPS+NS vs. control; ^#^*P*<0.05 LPS+NS vs. LPS+D-serine (n = 12/group). (D) Western blot analysis of NMDA receptor subunits (GluN2A, GluN2B, and GluN1) and synaptophysin in the hippocampus of surviving sepsis mice treated with normal saline or D-serine. Data are expressed as the mean ±S.D.*P<0.05 LPS+NS vs. LPS+D-serine for the given time point (n = 5 per time point per group).

D-serine is a co-agonist of synaptic NMDA receptors[17]. Thus, we detected the effects of the activation of NMDA receptors with D-serine on brain dysfunction in the surviving LPS-treated mice. In the open field test, there were no obvious differences in the total distance or the time spent in the central area among the normal control mice, the LPS +NS treated mice and the LPS+ D-serine treated mice [F(2,33) = 1.07, *P* = 0.35; Fig 2B]. In the Barnes maze test, the latency and errors to the target hole were obviously affected by the treatments [F(2,33) = 6.01, *P* = 0.03 for latency; F(2,33) = 4.01, *P* = 0.04 for errors] and time of measurements [F(3,99) = 4.36, *P* = 0.02 for latency; F(3,99) = 5.01, *P* = 0.02 for errors], but there was no significant effect of the interaction of treatment × measure time[F(6,99) = 1.30, *P* = 0.29 for latency; F(6,99) = 1.57, *P* = 0.21 for errors]. Further analysis showed that the latency to the target hole of the LPS+NS treated mice was significantly longer than that of the control or LPS+ D-serine treated mice on the 8th day and 9th day after LPS [8d: F(2,33) = 3.54, *P* = 0.03; 9d: F(2,33) = 4.44, *P* = 0.02; Fig 2C]. In addition, the errors of the LPS+NS treated mice were significantly more than those of the control or LPS+ D-serine treated mice on the 8th day, 9th day and 10th day after LPS [8d: F(2,33) = 4.59, *P* = 0.02; 9d: F(2,33) = 3.44, *P* = 0.02; 10d: F(2,33) = 3.19, *P* = 0.03; Fig 2C]. There was no obvious difference in the latency or errors between the control or LPS+ D-serine treated mice[8d: F(1,20) = 1.59, *P* = 0.08; 9d: F(1,20) = 1.74, *P* = 0.08; 10d: F(1,20) = 0.99, *P* = 0.13; Fig 2C]. Corresponding to the improvement of brain dysfunction, D-serine treatment limited the LPS-induced decrease in p-GluN2A/GluN2A,GluN2B,and GluN1levels in the hippocampus during the first 7days after LPS treatment (*P*<0.05).However, D-serine treatment did not affect the expression of synaptophysin (*P*>0.05; Fig 2D).

### Activation of NMDA receptors with D-serine obviously limited the neuroinflammation in the hippocampus of the surviving LPS-treated mice

**Fig 3:**
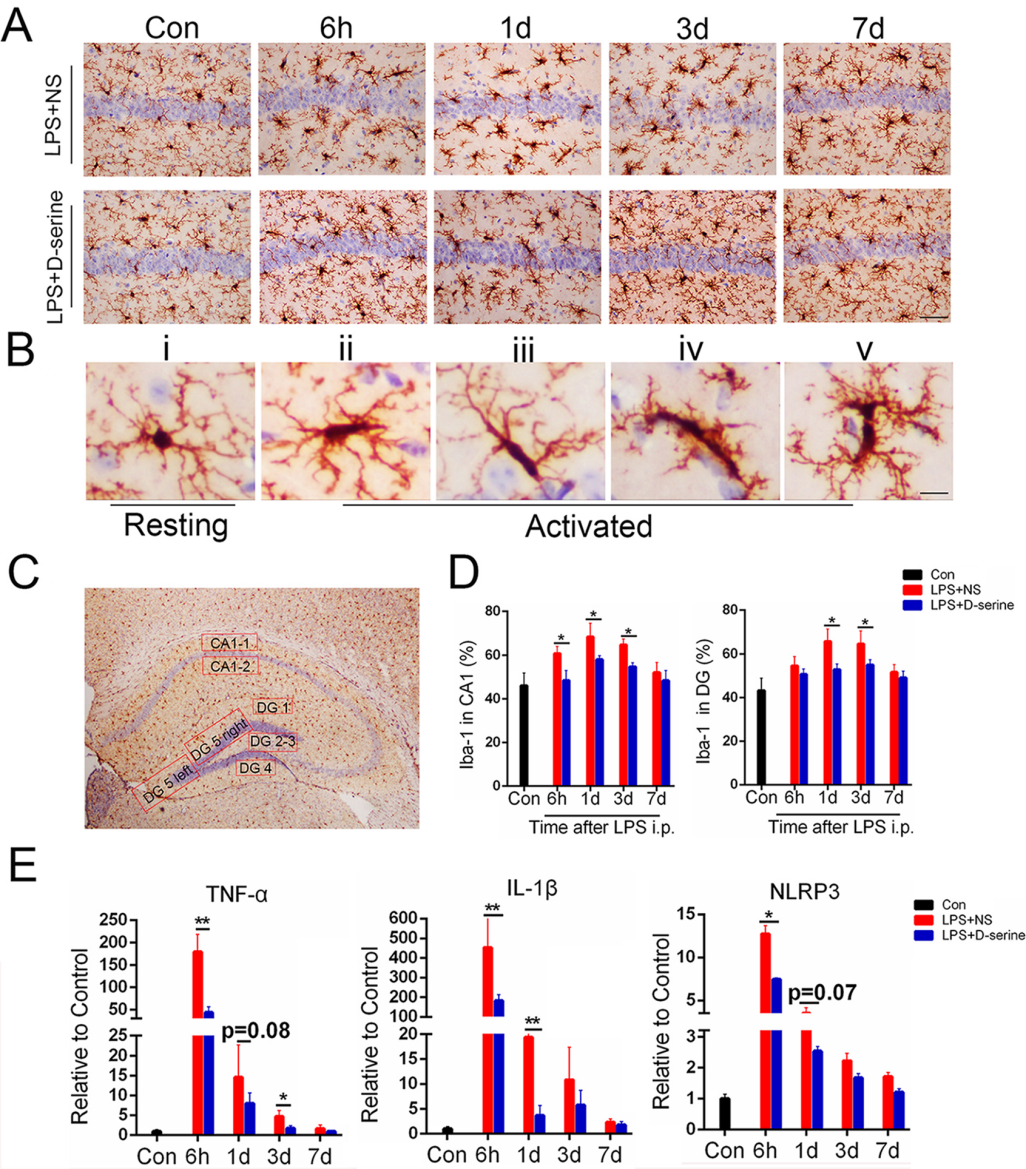
Activation of NMDA receptors with D-serine obviously limited neuroinflammation in the hippocampus of the surviving LPS-treated mice. (A) Representative images of Iba-1 staining in the CA1, Bar = 50μm. (B) Representative images of the resting microglia and different types of activated microglia, Bar = 200μm. (C) Representative selected regions of hippocampus used for counting the activated percentages of microglia. The mean of CA1-1 and CA1-2 represents the activated level of the CA1 region in each hippocampal section. The mean of DG1~DG5 represents the activated level of the DG region in each hippocampal section. (D) Representative statistical results of activated microglia percentages in the CA1 and DG regions. Data are expressed as the mean ±S.D.*P<0.05 LPS+NS vs. LPS+D-serine for the corresponding time point (n = 5 per time point per group). (E) Quantitative RT-PCR analysis of mRNA levels of inflammatory factors (TNF-α, IL-1β, and NLRP3) in the hippocampus. Data are expressed as the mean ±S.D.*P<0.05 and **P<0.01 LPS+NS vs. LPS+D-serine for the corresponding time point (n = 5 per time point per group).

Neuroinflammation is an important pathological mechanism of sepsis-related brain dysfunction[30]. Therefore, we evaluated hippocampal neuroinflammation by detecting the microglia activation and the mRNA levels of the inflammatory factors TNF-α, IL-1β, and NLRP3. Compared to the control mice, microglia were obviously activated in the LPS+NS group during the first 3days after LPS injection(Fig 3A and 3B). In addition, the morphological changes in the microglia in the LPS-D-serine group were less obvious than those in the LPS+NS group (Fig 3A and 3B). Further analysis showed that the percentages of microglia activation in the dentate gyrus and CA1 of the surviving LPS-treated mice were obviously increased during the first 3 days after LPS injection[DG:F(4,32) = 4.71, *P*<0.02;CA1: F(4,32) = 5.03, *P*<0.02; Fig 3D] compared to the normal control mice. Similarly, the mRNA levels of the inflammatory factors TNF-α, IL-1β, and NLRP3 were all increased in the hippocampus of the surviving LPS-treated mice during the first 3 days after surgery [TNF-α:F(4,32) = 10.47, *P*<0.001; IL-1β: F(4,32) = 12.33, *P*<0.001; NLRP3: F(4,32) = 8.12, *P*<0.001; Fig 3E]. However, the D-serine treatment obviously limited the LPS-induced microglia activation[DG: F(1,8) = 3.63, *P*<0.03;CA1: F(1,8) = 4.03,*P*<0.02] and the increased mRNA levels of the inflammatory factors TNF-α, IL-1β, and NLRP3 [TNF-α:F(1,8) = 4.19, *P* = 0.01; IL-1β: F(1,8) = 6.33, *P* = 0.01; NLRP3: F(1,8) = 3.12, *P* = 0.04; Fig 3D]. These data suggest that the activation of NMDA receptors by D-serine obviously limited hippocampal neuroinflammation in the surviving LPS-treated mice.

### Activation of NMDA receptors with D-serine obviously limited the decrease of p-CREB in the hippocampus of surviving LPS-treated mice

**Fig 4:**
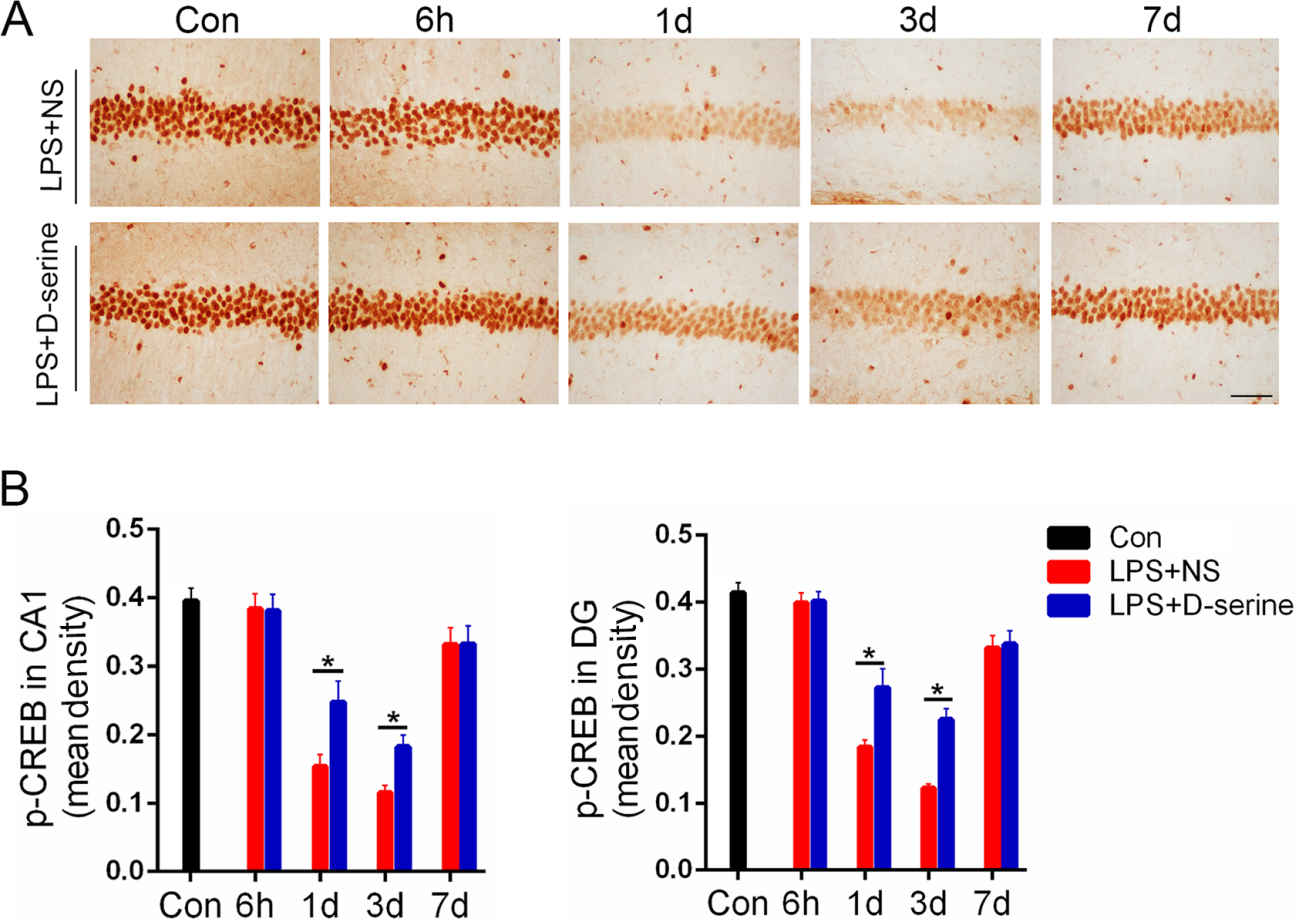
Activation of NMDA receptors with D-serine obviously limited the decrease of p-CREB in the hippocampus of the surviving LPS-treated mice. (A) Images of the p-CREB staining, Bar = 50μm. (B) The relative mean optical densities for p-CREB staining in the CA1 and dentate gyrus. Data are expressed as the mean ±S.D.*P<0.05 LPS+NS vs. LPS+D-serine for corresponding time points after LPS i.p. (n = 5 per time point per group).

Compared to the normal control mice, the relative mean optical density of p-CREB staining in the CA1and dentate gyrus of the surviving LPS-treated mice was obviously decreased on the 1st, 3rd,and 7th days after LPS treatment [1st:F(4,32) = 3.21, *P* = 0.03; 3rd: F(4,32) = 4.41, *P* = 0.02; 7th: F(4,32) = 2.31, *P* = 0.04; Fig 4]. However, D-serine treatment significantly limited the decrease of the relative mean optical density of p-CREB staining in the surviving LPS-treated mice on the 1st and 3rd days after LPS treatment [1st:F(1,8) = 3.19, *P* = 0.03; 3rd: F(1,8) = 4.33, *P* = 0.02; Fig 4].

### Activation of NMDA receptors with D-serine obviously inhibited the increase of ROS in the hippocampus of the surviving LPS-treated mice

**Fig 5:**
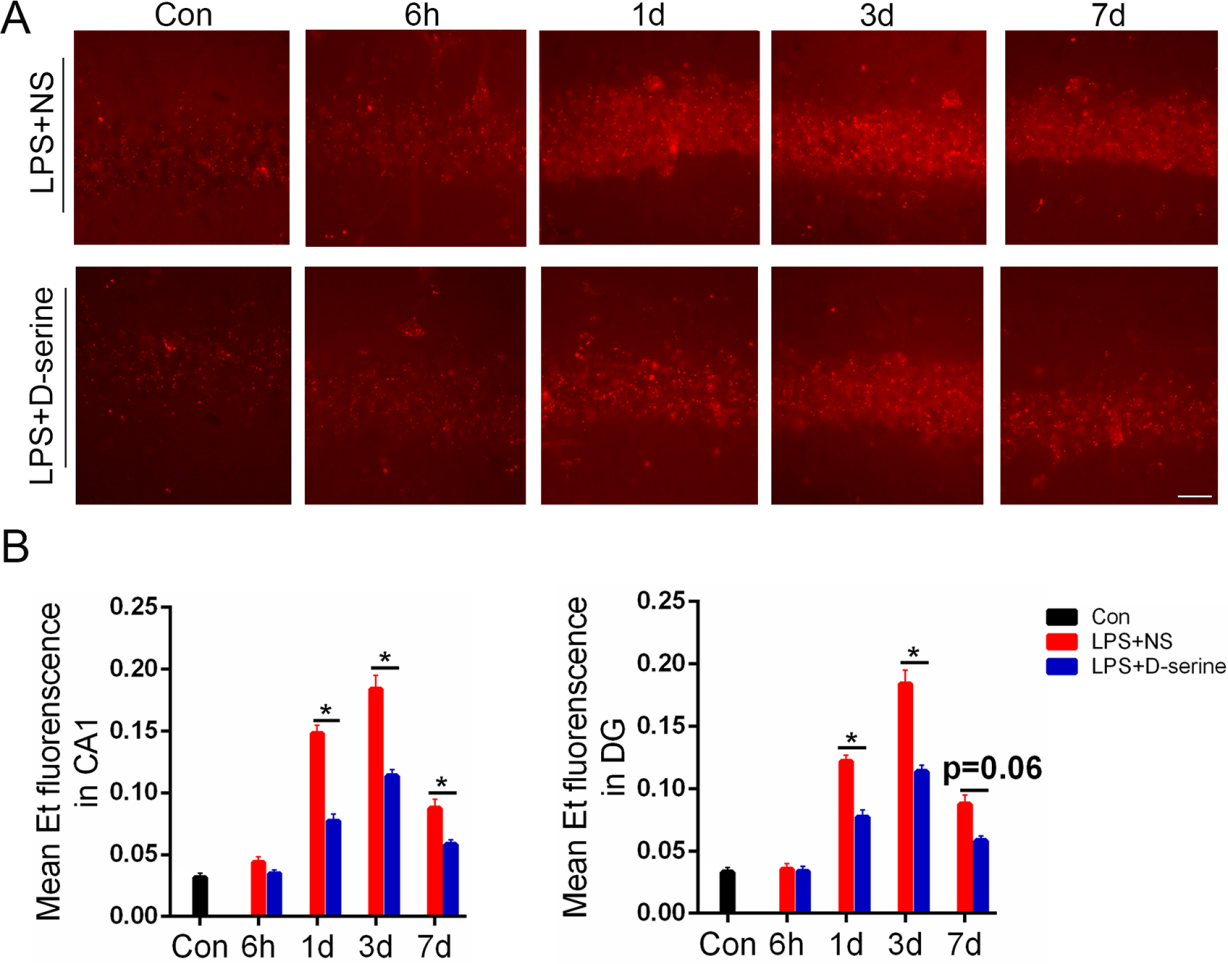
Activation of NMDA receptors with D-serine obviously inhibited the increase of ROS in the hippocampus of the surviving LPS-treated mice. (A) Representative images of ROS fluorescence in the CA1, Bar = 100μm. (B) Representative relative mean Et fluorescence in the CA1 and dentate gyrus, *P<0.05 LPS+NS vs. LPS+D-serine for corresponding time points. Data are expressed as the mean ±S.D. (n = 5 per time point per group).

Compared to the normal control mice, the ROS levels in the CA1 and dentate gyrus of the surviving LPS-treated mice was obviously increased on the 1st,3rd, and 7th days after LPS treatment [1st:F(4,32) = 3.18, *P* = 0.02; 3rd: F(4,32) = 4.13, *P* = 0.01; 7th: F(4,32) = 2.59, *P* = 0.03; Fig 5]. D-serine treatment significantly inhibited the increase in ROS levels in the surviving LPS-treated mice on the 1st, 3rd, and 7th days after LPS treatment [1st:F(1,8) = 3.19,*P* = 0.03; 3rd: F(1,8) = 2.39, *P* = 0.04; 7th: F(1,8) = 1.93, *P* = 0.04; Fig 5].

## Discussion

Our aims were to investigate the expression levels and roles of NMDA receptor subunits during sepsis-related brain dysfunction. We found that 1) sepsis selectively decreased the levels of GluN2A, GluN2B andGluN1 but not synaptophysin or neuron number in the hippocampus of mice during the first 7days after sepsis; and 2) the NMDA receptor co-agonist D-serine significantly improved the brain dysfunction and the neuropathology of the hippocampus in septic survivors. These results suggest that NMDA receptors are closely involved with the brain dysfunction of septic survivors and are potential targets for improvement of sepsis-related brain dysfunction.

Many studies have shown that sepsis-induced neuroinflammation plays an important role in sepsis-related brain dysfunction[1]. However, the molecules that are targeted during neuroinflammation resulting from sepsis-induced brain dysfunction remain unclear. NMDA receptors are closely involved with synaptic plasticity, learning and memory[14]. Thus, we detected the expression of NMDA receptor subunits in the hippocampus in two classic sepsis models: the LPS model and the CLP model. We found that sepsis selectively decreased the levels of GluN2A, GluN2B and GluN1 in the hippocampus of septic mice. To further confirm the selectivity of the loss of sepsis-induced NMDA receptor expression, we also detected the expression of the synaptic protein synaptophysin and the neuron number in the CA1. There were no obvious changes in synaptophysin expression in the hippocampus or in the numbers of neuron in the CA1 of septic mice, which were detected in the two classic sepsis models. These data show that sepsis selectively induced the loss of NMDA receptor subunits. In agreement with our data, Ma J also found that chronic neuroinflammation induced by chronic infusion of LPS into the fourth ventricle decreased the levels of GluN2A and GluN2B in the hippocampus of Fischer-344 rats[31]. In addition, previous studies have reported that a small dose of LPS mainly affected the expression of GluN2B but not GluN1 or GluN2A in the first 28 hours of sepsis[15,16], which is obviously different from our results. The difference between our data and the data in previous studies possibly results from the difference in LPS dose and the time points of detection. However, the reason behind these contrasting results remains unclear.

D-serine is commonly used as a co-agonist of NMDA receptors, with much a higher affinity for synaptic NMDA receptors[17,32]. GluN2A is a main subunit of synaptic NMDA receptors[14,17]. Therefore, we used D-serine to account for the loss of NMDA receptor subunits during sepsis, and then detected the role of the selective loss of NMDA receptors in sepsis-related brain dysfunction. Interestingly, sepsis resulted in an obvious impairment of learning and memory in the mice, in microglia activation, in inflammatory factor expression, in ROS stress, and in the decrease of neuronal activity marked by p-CREB (Figs 2, 3, 4 and 5). However, these changes induced by sepsis were all partly reversed by D-serine (administered once per day; Figs 2, 3, 4 and 5).These data are consistent with the report ofFarooq A[33]. They showed that activation of NMDAR could downregulate inflammasomal activity and inhibit inflammation in the liver[33]. In addition, chronic neuroinflammation could decreased the levels of GluN2A and GluN2B in the hippocampus[31]. Thus, there is possibly a vicious circle between NMDA receptor loss and neuroinflammation, which results in sepsis-related brain dysfunction. In addition, previous studies have shown that HMGB1, an important danger signal molecule, is closely involved in sepsis via Toll-like receptors[34,35]. In addition, Balosso et al found that Toll-like receptors and NMDA receptor subunits are co-localized in neurons. Our previous study showed that blocking HMGB1 with a specific anti-HMGB1 antibody could modulate the expression of GluN2A and GluN2B in the hippocampus of aged rats after surgery[25]. These results suggest that HMGB1 possibly plays a role in the modulation of the selective loss of NMDA receptors during sepsis-induced neuroinflammation.

In the present study, we have not detected the effect of D-serine on brain dysfunction or neuropathology in the CLP model. There are two reasons. First, the LPS model and the CLP model can both induce classic sepsis-related neuropathology including a selective loss of NMDA receptors (Fig 1). Second, LPS model is more homogeneous than the CLP model, which is helpful to guarantee the reproducibility of our data. Taken together, our data show that sepsis can induce the selective loss of NMDA receptors, which modulate the neuropathology of sepsis-related brain dysfunction.

## Supporting information

S1 File ARRIVE guidelines checklist. (DOCX)
