# Supplementary material for: Sepsis-induced selective loss of NMDA receptors modulates hippocampal neuropathology in surviving septic mice
Source: PLoS One. 2017 Nov 27;12(11):e0188273. doi: 10.1371/journal.pone.0188273 (PMC5703474; doi:10.1371/journal.pone.0188273)
Supplement: S1 File — (DOCX) [file pone.0188273.s001.docx]

**ARRIVE guidelines checklist**

**Items**

1. **Title**

Sepsis-induced selective loss of NMDA receptors modulates hippocampal neuropathology in surviving septic mice

**2. Abstract**

Sepsis-induced neuroinflammation plays an important role in sepsis-related brain dysfunction. However, the molecules that are targeted during neuroinflammation resulting from sepsis-induced brain dysfunction remain unclear. Herein, we tried to investigate the expression and roles of NMDA receptor subunits during sepsis-related brain dysfunction. Sepsis was induced by cecal ligation and perforation (CLP) or by a single intraperitoneal injection of lipopolysaccharide (LPS, 8 mg/kg) in C57BL/6J mice. The NMDA receptor co-agonist D-serine was injected intraperitoneally for 3 days (500 mg/kg/day) to compensate for the loss of NMDA receptors. The behaviors of mice were tested in the Barnes maze and in the open field test. The mice were euthanized at the indicated time points. The brains were collected to detect the following: the levels of synaptophysin and NMDA receptor subunits GluN2A, GluN2B and GluN1 (by Western blot and RT-PCR); the number of CA1 neurons (by Nissl staining); neuronal activity (by p-CREB staining); neuroinflammation (by staining of Iba-1 and inflammatory factors IL-1β, TNF-α, NLRP3); and the levels of oxidative stress [by dihydroethidium (DHE)]. Sepsis selectively decreased the protein and mRNA levels of GluN2A, GluN2B and GluN1 but not the levels of synaptophysin or the neuronal number in the hippocampus of mice in either of the classic CLP-induced or LPS-induced sepsis models during the first 7 days after sepsis. Intraperitoneal injection of D-serine obviously limited the lipopolysaccharide-induced changes, including the impairment of learning and memory, the loss of NMDA receptor subunits, robust neuroinflammation, the levels of ROS stress and the decrease of p-CREB in the hippocampus of mice. These data suggest that the sepsis-induced selective loss of NMDA receptors modulates hippocampal neuropathology in the mice that survived sepsis, and the data show that NMDA receptors are potential targets for the improvement of brain dysfunction in sepsis survivors.

**Introduction**

1. **Background**

Sepsis-induced neuroinflammation plays an important role in sepsis-related brain dysfunction(J Clin Invest. 2016 Jan;126(1):23-31). However, the molecules that are targeted during neuroinflammation resulting from sepsis-induced brain dysfunction remain unclear. NMDA receptors are ionotropic glutamate receptors, which are closely involved in synaptic plasticity, learning and memory(Neuroscientist. 2013 Feb;19(1):62-75. ). Previous studies have detected the changes of NMDA receptors at the early stage of sepsis, and have shown that NMDA receptors could be the molecular targets of sepsis damage, which were involved in the sepsis-related brain dysfunction in septic survivors(Mol Brain. 2014 Apr 24;7:33, Brain Behav Immun. 2016 Feb;52:120-131.). However, both the changes of NMDA receptors at the late stage of sepsis and the roles of NMDA receptors in sepsis-related brain dysfunction still remain unclear. Herein, we tried to investigate the expression and roles of NMDA receptor subunits during sepsis-related brain dysfunction.

1. **Objective**

To investigate the expressions and roles of NMDA receptor subunits during the sepsis-related brain dysfunction.

**Methods**

1. **Ethical statement**

Experiments were performed in accordance with the guidelines for experimental animal use of Central South University. The protocol [LLSC (LA).2015-018] was approved by the ethics committee of the 3rd Xiangya Hospital of Central South University.

1. **Study design**

C57BL/6J male mice (2 months, 20-25 g) were purchased from Central South University（Hunan, China）. The experiments included two parts. Experiment 1 was used to detect the effects of sepsis on the expressions of NMDA receptors at the late stage of sepsis. Experiment 2 was used to detect the roles and underlying mechanisms of NMDA receptor subunits during the sepsis-related brain dysfunction.

Experiment 1:

C57BL/6J mice

CLP model (n=31)

LPS model (n=25)

1. NMDA receptor expressions of hippocampus were detected by PCR and Western blot.
2. Neuron numbers of hippocampus were detected by Nissl staining

Experiment 2 :

C57BL/6J mice

LPS+D-serine（500mg/kg for 3 days）（n=74）

LPS+NS (N=74)

Con (n=22)

1. Detecting behavioral changes by open field test( on day 7 after LPS) and Barnes maze test( on day 8-11 after LPS).
2. Detecting the levels of inflammatory factors(TNF-α, IL-1β and NLRP3) of hippocampus by PCR;
3. Detecting the levels of NMDA receptor subunits and synaptophysin by western blot;
4. Detecting microglia activation by Iba-1 staining, neuronal activity by p-CREB staining and ROS level by Dihydroethidium.

**7. Experimental procedures:**

LPS (8 mg/kg, once per mouse, Sigma-Aldrich, America, L2880) was diluted with normal saline and was intraperitoneally injected. D-serine (500 mg/kg, Sigma-Aldrich, America, S4250) was prepared with normal saline and was intraperitoneally injected for three days after LPS injection. All animals are sacrificed under sevoflurane anesthesia to eliminate the suffering.

1. **Experimental animals:**

Male C57BL/6J mice (2 months, 20-25 g)

1. **Housing and husbandry:**

All animals were housed with a 12-hour day/night cycle with free access to food and water in a temperature-controlled room at 24±1 °C with specific pathogen free（SPF）. All mice were acclimated to the environment for seven days before the experiments.

| 1. **Sample size:**   According to our study design, we used fifty-six mice in experiment 1,and one hundred seventy mice in experiment 2 and 3.   1. **Allocating animals to experimental groups:**  \| All animals are randomly divided into control group and drug or surgery-treated group by computer-based randomized grouping. \| \| --- \| |
| --- | --- |

1. **Experimental outcomes:**

Sepsis selectively decreased the protein and mRNA levels of GluN2A, GluN2B and GluN1 but not the levels of synaptophysin or the neuronal number in the hippocampus of mice in either of the classic CLP-induced or LPS-induced sepsis models during the first 7 days after sepsis. Intraperitoneal injection of D-serine obviously limited the lipopolysaccharide-induced changes, including the impairment of learning and memory, the loss of NMDA receptor subunits, robust neuroinflammation, the levels of ROS stress and the decrease of p-CREB in the hippocampus of mice.

1. **Statistical methods:**

The Barnes maze data are presented as the mean ± standard error (mean ± SEM) and were analyzed using repeated measures ANOVA with the treatment as the between-subjects factor and the measure of time as the within-subjects factor, followed by the Bonferroni test. Biochemical data are presented as the mean ± standard deviation (mean ± SD) and were analyzed using two-way ANOVA followed by the Bonferroni test. Data analyses were performed using SPSS 18.0 for Windows, and P<0.05 was considered statistically significant.

**Results:**

1. **Baseline data:**

For each experiment group, the weight，mental state and activity are at the same level or there was no statistical significance between each group prior to each testing or treatment.

1. **Numbers analysed:**

Animal number sizes were estimated by PASS software , based on the preliminary data.

1. **Outcomes and estimation:**

Data analyses were performed using SPSS 18.0 for Windows, and P<0.05 was considered statistically significant.

1. **Adverse events:**

It is very important to keep body temperature and fluid resuscitation of mice during the CLP model.

**Discussion**

1. **Interpretation/scientific implications:**

Our data suggest that sepsis-induced selective loss of NMDA receptors modulates the neuropathology of hippocampus in the surviving mice of sepsis, and is a potential target for improvement of the brain dysfunction of septic survivors.

1. **Generalisability/translation:**

Targeting NMDA receptors may be useful for the prevention and treatment of Sepsis associated encephalopathy.

1. **Funding:**

This study was supported by the grants from the National Natural Science Foundation of China (No.81371216, No.81471107 and No.81400896) .Jianbin Tong and Yuan Le are co-corresponding authors of this study, and Wen Ouyang is an important partner of this study.
